# Impact of socioeconomic deprivation on the rate of residual or recurrent cholesteatoma following primary mastoidectomy

**DOI:** 10.1017/S002221512610499X

**Published:** 2026-07

**Authors:** Jane Fang Foo, Kerry Haddow, Stephen Jones, Patrick Spielmann

**Affiliations:** ENT Department, Ninewells Hospital and Medical School, University of Dundee, Dundee, Scotland

**Keywords:** adult, child, cholesteatoma, health status disparities, recurrence, socioeconomic factors

## Abstract

**Objectives:**

To investigate whether socioeconomic deprivation is associated with residual or recurrent cholesteatoma following primary mastoidectomy.

**Methods:**

A retrospective cohort study included all patients undergoing primary mastoidectomy for cholesteatoma in National Health Service Tayside between 2012 and 2020. Socioeconomic status was assessed using the Scottish Index of Multiple Deprivation 2020 and divided into quintiles. Patients were stratified into paediatric (≤16 years) and adult (>16 years) groups. Recurrence/residual rates across Scottish Index of Multiple Deprivation quintiles were compared using Fisher’s exact tests, and time to recurrence was analysed using Kaplan–Meier survival curves.

**Results:**

One hundred eighty-four patients were included (59 children, 125 adults). Recurrence/residual occurred in 39 per cent of children and 24 per cent of adults. In children, higher deprivation was significantly associated with risk of recurrence/residual disease (*p* = 0.0013). No significant association was observed in adults.

**Conclusion:**

Socioeconomic deprivation is linked to higher recurrence/residual in children, highlighting disparities in postoperative outcomes and the importance of targeted follow-up.

## Introduction

Cholesteatoma is an abnormal collection of keratinising squamous epithelium within the middle ear and mastoid, capable of causing local bone erosion through enzymatic activity and chronic inflammation.^1^ Surgical excision remains the mainstay of management. Nevertheless, cholesteatoma is associated with considerable rates of residual and recurrent disease. Distinguishing between these two can sometimes be challenging. Reported recurrence rates vary widely across studies.^2^ A large study conducted in a Danish population reported a 5-year recurrence rate of 37 per cent in children and 15 per cent in adults.^3^ Numerous studies have looked at factors associated with recurrence, including disease location, extent of ossicular erosion, surgical approach and technique and patient-related characteristics such as age, Eustachian tube dysfunction and comorbidities.^2^

There is well documented evidence showing that broader social determinants of health may influence disease burden and outcomes.^4^ Several population-based studies have demonstrated a higher incidence of cholesteatoma in socioeconomically deprived groups, with these patients often presenting later and with more extensive disease. Although this association with disease incidence and severity is well recognised, it remains uncertain whether socioeconomic deprivation similarly affects recurrence rates following primary mastoid surgery for cholesteatoma. Most studies have looked at anatomical and surgical factors that affect recurrence, but there is paucity of data on the impact of socioeconomic status (SES). This represents an important gap in the literature, as deprivation can influence access to specialist care, follow-up and overall health outcomes.^5^

The Scottish Index of Multiple Deprivation (SIMD) is the Scottish Government’s standard measure of area-based deprivation.^6^ SIMD captures multiple aspects of deprivation, including income, employment, education, health, access to services, crime and housing, providing a broader picture of socioeconomic disadvantage in a given area. Scotland is divided into 6,976 areas, ranked from most deprived (Rank 1) to least deprived (Rank 6,976). These are then grouped into quintiles for analysis (Quintile 1: the 20 per cent most deprived; Quintile 5: the 20 per cent least deprived).

While SIMD reflects area-level deprivation rather than individual circumstances, it provides a robust framework to explore social determinants of health. In this study, conducted within the NHS Tayside health board region, we examine the association between SIMD quintile and rates of residual or recurrent cholesteatoma following primary mastoidectomy performed between 2012 and 2020. To our knowledge, this is one of the first cohort studies to specifically investigate whether socioeconomic deprivation, objectively measured by SIMD, is associated with residual or recurrent disease. In a retrospective study, it can be difficult to differentiate between residual and recurrent cholesteatoma, so we have grouped them together to capture the burden of cholesteatoma. The term recidivism is used to describe both residual and recurrent disease. Outcomes were stratified by adult and paediatric cohorts to explore potential age-related differences in disease behaviour and follow-up dynamics, providing further insight into post-operative outcomes.

## Materials and methods

This retrospective cohort study was carried out within the NHS Tayside health board and included all patients who underwent primary mastoidectomy for cholesteatoma between 2012 and 2020. Patients were initially identified via the local operative theatre coding system, then cross-referenced with the departmental theatre diary, and further verified through electronic health records and operative notes. Only cases with an intraoperatively confirmed diagnosis of cholesteatoma were included in the analysis. Patients were excluded if they had previously undergone mastoid surgery (i.e., revision cases), or if they had moved out of NHS Tayside during the follow-up period, resulting in no meaningful follow-up data available.

The primary outcome was the presence of residual or recurrent cholesteatoma following initial surgery. Residual disease was defined as persistent cholesteatoma detected during early postoperative imaging or unplanned re-exploration, likely representing incomplete clearance at the initial operation. Recurrent disease was defined as cholesteatoma arising after a period of confirmed disease-free status. Typical post-operative follow-up protocol for adults and children in our institution is three weeks, three months, 12 months and then 6–12 monthly depending on disease status. In more recent years, non–echo-planar diffusion-weighted magnetic resonance imaging (non-EPI DWI MRI) has increasingly been used as an alternative to planned second-look surgery, particularly in the paediatric population. Any cholesteatoma detected during postoperative surveillance, whether by non-EPI DWI MRI or unplanned surgical re-exploration, was classified as residual or recurrent, since complete clearance had been intended at the initial surgery. In rare cases where subtotal resection with a planned staged procedure was performed due to extensive disease or patient factors, cholesteatoma at the subsequent planned operation was expected and hence not considered residual or recurrent.

Each patient’s demographic information, comorbidities, operative details, intraoperative findings, disease characteristics and post-operative surveillance were recorded. Socioeconomic status was assessed using the SIMD 2020, based on the patient’s residential postcode at time of surgery. SIMD ranks were grouped into quintiles, with Quintile 1 representing the most deprived and Quintile 5 the least. Patients were stratified into paediatric (≤16 years at time of surgery) and adult (>16 years) cohorts at time of primary surgery. In cases of bilateral disease, residual/recurrent disease was assessed by ear, rather than by patient.

### Data analysis

Descriptive statistics were used to summarise baseline characteristics. Residual or recurrent disease rates were compared across SIMD quintiles and age groups using Fisher’s exact tests. Time to recurrence was analysed using Kaplan–Meier survival analysis. Logistic regression modelling was planned to assess the independent association between SIMD and recurrence risk, adjusting for potential confounders including age, smoking status, surgical technique and extent of disease based on intra-operative findings. A two-sided *p*-value of less than 0.05 was considered statistically significant. All statistical analyses were performed using Microsoft Excel for Mac Version 16.87, IBM SPSS Statistics Version 30.0.0.0 (172) and GraphPad Prism 10 for macOS Version 10.6.1 (799).

## Results and analysis

From an initial cohort of 375 patients, 191 were excluded based on the above exclusion criteria, leaving a final sample size of 184 patients for analysis. Thirteen patients were lost to follow-up or moved to a different health board. The other patients were excluded as the surgery was a revision case. Of the 184 patients, 59 were children and 125 were adults.

### Children (≤16)

**Figure 1.**
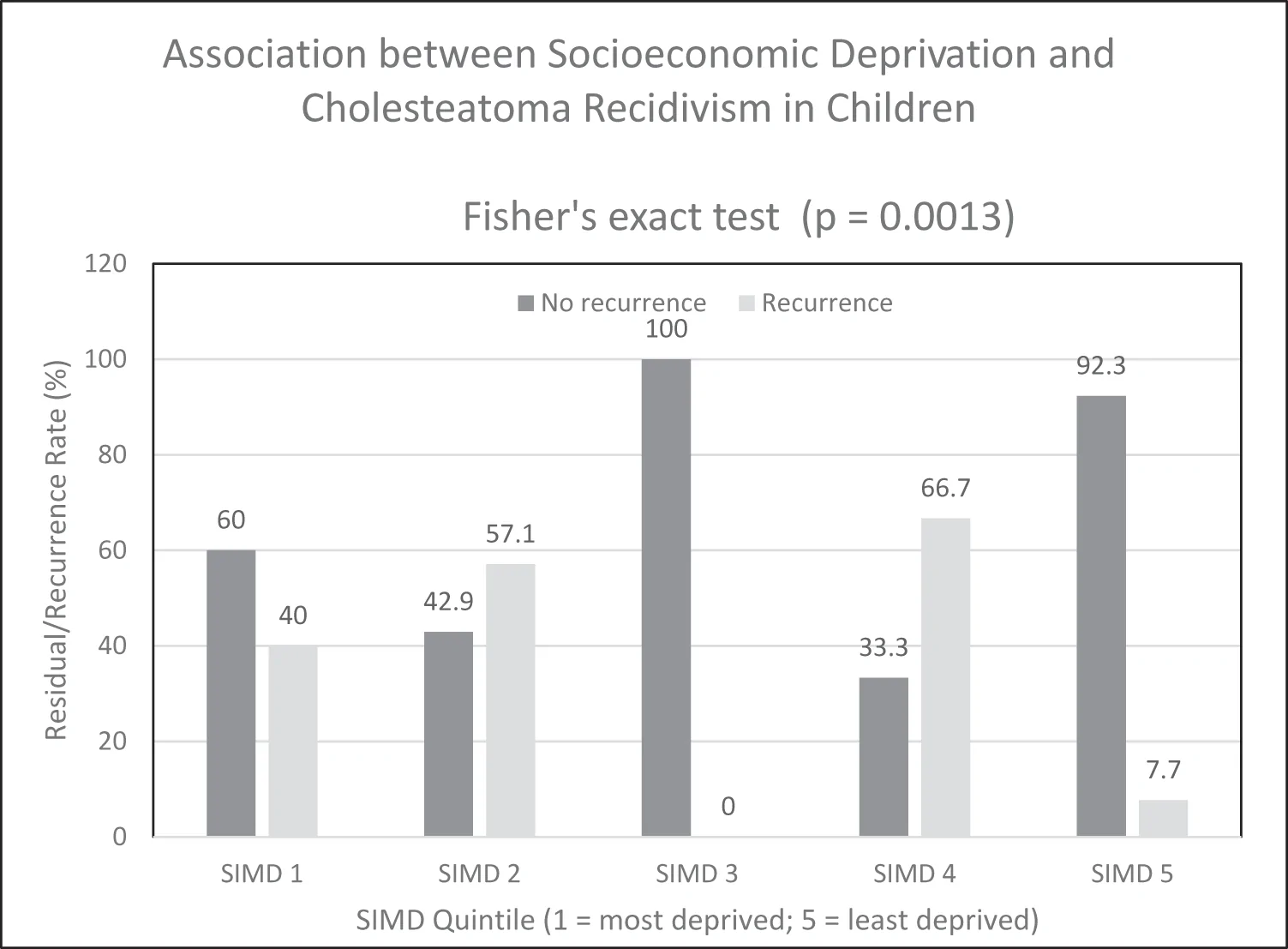
Residual or recurrent rate of paediatric cholesteatoma by SIMD quintile. Figure 1 long description.

A total of 59 paediatric patients were included in the analysis. Baseline characteristics, surgical factors and post-operative follow-up are summarized in Table 1. Bilateral disease was present in 13.8 per cent of children, and the overall residual/recurrence rate was 23 of 59 patients (39.0 per cent; 95 per cent confidence interval [CI]: 26.5–52.6). A breakdown of residual/recurrence rate by SIMD quintile is shown in Figure 1. Associations between categorical variables and recidivism were assessed using Fisher’s exact test, which demonstrated a significant association between SIMD quintiles and recurrence (*p* = 0.0013).

**Table 1.** Baseline patient characteristics, surgical factors and post-operative follow-up in children Table 1 long description.

Parameter	Overall	SIMD 1	SIMD 2	SIMD 3	SIMD 4	SIMD 5
**Patient demographics**						
Total	59	10	14	7	15	13
Gender						
Male	35 (59.3%)	4 (40.0%)	7 (50.0%)	6 (85.7%)	12 (80.0%)	6 (46.2%)
Female	24(40.7%)	6 (60.0%)	7 (50.0%)	1 (14.3%)	3 (20.0%)	7 (53.8%)
**Disease and surgical factors**						
Surgical Technique						
Canal wall-down	11 (18.6%)	2 (20.0%)	3 (21.4%)	1 (14.3%)	4 (26.7%)	1 (7.7%)
Canal wall-up	48 (81.4%)	8 (80.0%)	11 (78.6%)	6 (85.7%)	11 (73.3%)	12 (92.3%)
Location of primary disease						
Attic	39 (66.1%)	8 (80.0%)	8 (57.1%)	4 (57.1%)	10 (66.7%)	9 (69.2%)
Pars tensa	14 (23.7%)	2 (20.0%)	4 (28.6%)	3 (42.9%)	2 (13.3%)	3 (23.1%)
Both	6 (10.2%)	0	2 (14.3%)	0	3 (20.0%)	1 (7.7%)
Extension into mastoid antrum						
No	12 (20.3%)	3 (30.0%)	0	3 (42.9%)	3 (20.0%)	3 (23.1%)
Yes	47 (79.7%)	7 (70.0%)	14 (100.0%)	4 (57.1%)	12 (80.0%)	10 (76.9%)
Ossicular chain erosion						
No	10 (16.9%)	0 (0.0%)	3 (21.4%)	0 (0.0%)	3 (20.0%)	4 (30.8%)
Yes	49 (83.1%)	10 (100.0%)	11 (78.6%)	7 (100.0%)	12 (80.0%)	9 (69.2%)
Dehiscent facial nerve						
No	57 (96.6%)	8 (80.0%)	14 (100.0%)	7 (100.0%)	15 (100.0%)	13 (100.0%)
Yes	2 (3.4%)	2 (20.0%)	0	0	0	0
**Post-operative**						
Recurrence after primary surgery						
Yes	23 (39.0%)	4 (40.0%)	8 (57.1%)	0 (0%)	10 (66.7%)	1 (7.7%)
No	36 (61.0%)	6 (60.0%)	6 (42.9%)	7 (100%)	5 (33.3%)	12 (92.3%)
Median follow-up time, months (IQR)	74.0	66.5	84.5	58.0	101.0	48.0
	(45.5 – 103.0)	(41.8 – 105.5)	(51.5 – 95.3)	(45.0 – 69.5)	(70.0 – 114.0)	(33.0 – 85.0)
Planned second-look surgery done	24/59 (40.7%)	6/10 (60.0%)	1/14 (7.1%)	4/7 (57.1%)	8/15 (53.3%)	6/13 (46.2%)
No disease	10	2	0	4	1	3
Recurrence	5	2	0	0	3	1
Residual	9	2	1	0	4	2
Postop MRI done	38/59 (64.4%)	3/10 (30.0%)	10/14(71.4%)	5/7 (71.4%)	12/15 (80.0%)	8/13 (61.5%)
No evidence of disease on MRI	24	2	4	5	5	8
Evidence of disease on MRI	14	1	6	0	7	0

**Figure 2.**
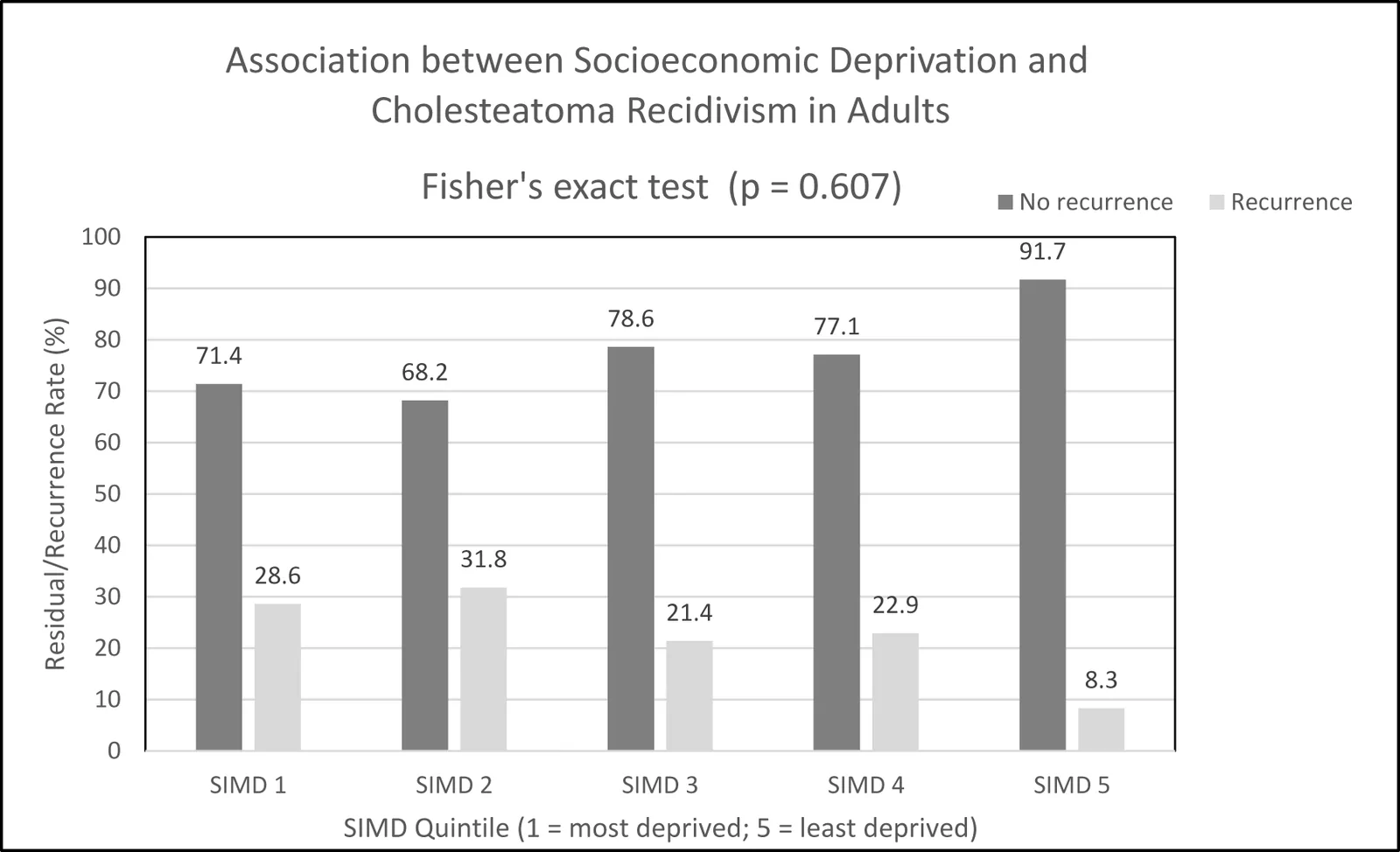
Residual or recurrent rate of adult cholesteatoma by SIMD quintile. Figure 2 long description.

Kaplan–Meier analysis of residual/recurrence-free time in children revealed a significant difference between SIMD quintiles (log-rank [Mantel–Cox] χ^2^ = 13.05; df = 4; *p* = 0.011) (Figure 2).

Location of primary disease and extension into the mastoid antrum were significantly associated with residual/recurrence rate. Children with disease involving both attic and pars tensa had the highest residual/recurrence rate (100 per cent), followed by attic-origin disease alone (33.3%) and pars tensa-origin disease (28.6 per cent) (χ^2^ = 10.55; *p* = 0.005). Similarly, mastoid antrum extension was associated with higher recidivism (*p* = 0.005). Notably, SIMD quintile was not associated with disease origin (χ^2^ = 5.995; *p* = 0.648) or mastoid antrum extension (χ^2^ = 6.558; *p* = 0.161).

On univariate analysis, none of the other examined variables (gender, age at surgery and canal wall status [canal wall up, CWU vs. canal wall down, CWD], ossicular chain erosion or dehiscent facial nerve) were significantly associated with recidivism. Multivariate logistic regression could not be reliably performed due to small sample size and perfectly predictive categories, so associations are reported based on univariate analyses.

### Adults (>16)

**Figure 3.**
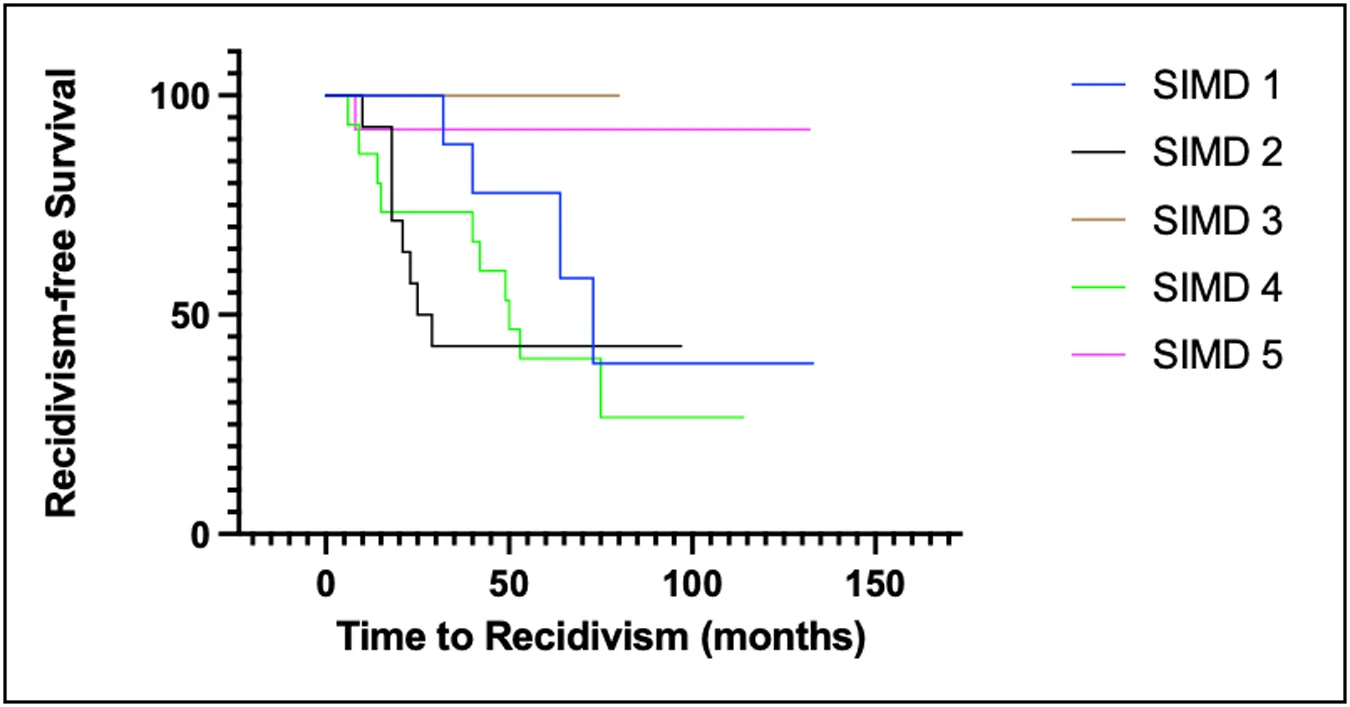
Kaplan–Meier analysis of recidivism over time in children (log-rank [Mantel–Cox] χ^2^ = 13.05, df = 4, p = 0.011). Figure 3 long description.

**Figure 4.**
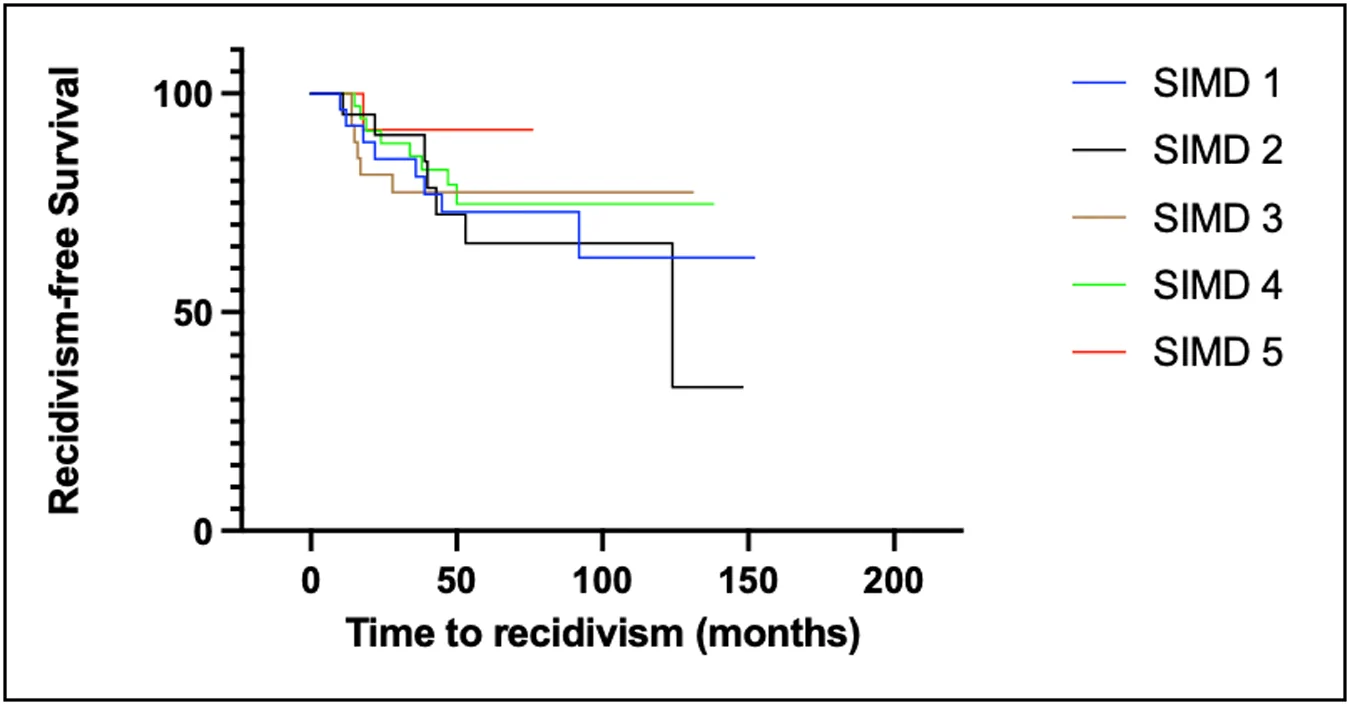
Kaplan–Meier analysis of recidivism over time in adults (Log-rank [Mantel–Cox] χ^2^ = 1.86, df = 4, p = 0.76). Figure 4 long description.

A total of 125 adult patients were included in the analysis. Baseline patient characteristics, surgical factors and post-operative follow-up details are summarized in Table 2. The overall residual/recurrence rate in adults was 30 out of 125 patients (24.0 per cent; 95 per cent CI: 16.8–32.5 per cent). Bilateral disease was present in 9.5 per cent of adults. Associations between categorical variables and recurrence were also assessed using Fisher’s exact test, which did not demonstrate any significant associations (p = 0.622). A breakdown of residual/recurrence rate by SIMD quintile is shown in Figure 3. Kaplan–Meier analysis did not demonstrate a significant difference in recidivism over time (Log-rank [Mantel–Cox] χ^2^ = 1.86; df = 4; *p* = 0.76) (Fig. 4).

**Table 2.** Baseline patient characteristics, surgical factors and post-operative follow-up in adults Table 2 long description.

Parameter	Overall	SIMD 1	SIMD 2	SIMD 3	SIMD 4	SIMD 5
**Patient demographics**						
Total	125	28	22	28	35	12
Gender						
Male	64 (51.2%)	14 (50.0%)	11 (50.0%)	9 (32.1%)	22 (62.9%)	8 (66.7%)
Female	61 (48.8%)	14 (50.0%)	11 (50.0%)	19 (67.9%)	13 (37.1%)	4 (33.3%)
Smoking status						
Smoking (at time of surgery)	38 (30.4%)	11 (39.3%)	9 (40.9%)	6 (21.4%)	11 (31.4%)	1 (8.3%)
Lifelong non-smoker	81 (64.8%)	17 (60.7%)	11 (50.0%)	20 (71.4%)	23 (65.7%)	10 (83.3%)
Ex-smoker	6 (4.8%)	2 (7.1%)	2 (9.1%)	2 (7.1%)	1 (2.9%)	1 (8.3%)
**Disease and surgical factors**						
Surgical technique						
Canal wall-down	87 (69.6%)	15 (53.6%)	17 (77.3%)	21 (75.0%)	26 (74.3%)	8 (66.7%)
Canal wall-up	38 (30.4%)	13 (46.4%)	5 (22.7%)	7 (25.0%)	9 (25.7%)	4 (33.3%)
Location of primary disease						
Attic	106 (84.8%)	24 (85.7%)	20 (90.9%)	22 (78.6%)	30 (85.7%)	10 (83.3%)
Pars tensa	14 (11.2%)	4 (14.3%)	2 (9.1%)	3 (10.7%)	3 (8.6%)	2 (16.7%)
Both	5 (4.0%)	0	0	3 (10.7%)	2 (5.7%)	0
Extension into mastoid antrum						
No	42 (33.6%)	9 (32.1%)	8 (36.4%)	11 (39.3%)	8 (22.9%)	6 (50.0%)
Yes	83 (66.4%)	19 (67.9%)	14 (63.6%)	17 (60.7%)	27 (77.1%)	6 (50.0%)
Ossicular chain erosion						
No	34 (27.2%)	7 (25.0%)	6 (27.3%)	7 (25.0%)	10 (28.6%)	4 (33.3%)
Yes	91 (72.8%)	21 (75.0%)	16 (72.7%)	21 (75.0%)	25 (71.4%)	8 (66.7%)
Dehiscent facial nerve						
No	101 (80.8%)	25 (89.3%)	18 (81.8%)	21 (75.0%)	27 (77.1%)	10 (83.3%)
Yes	24 (19.2%)	3 (10.7%)	4 (22.2%)	7 (25.0%)	8 (22.9%)	2 (16.7%)
**Post-operative**						
Recurrence after primary surgery						
Yes	30 (24.0%)	8 (28.6%)	7 (31.8%)	6 (21.4%)	8 (22.9%)	1 (8.3%)
No	95 (76.0%)	20 (71.4%)	15 (68.2%)	22 (78.6%)	27 (77.1%)	11 (91.7%)
Median follow-up time, months (IQR)	60.0	74.5	56.5	61.5	63.0	44.5
	(47.0 – 87.0)	(50.3 – 93.5)	(49.8 – 67.0)	(48.5 – 81.8)	(48.5 – 94.5)	(38.3 – 58.0)
Planned second-look surgery done	9/125 (7.2%)	5/28 (17.9%)	0/22 (0%)	2/28 (7.1%)	1/35 (2.9%)	1/12 (8.3%)
No disease	0	0	–	0	0	0
Recurrence	2	2	–	2	0	0
Residual	7	3	–	0	1	1
Postop MRI done	26/125 (20.8%)	6/28 (21.4%)	3/22 (13.6%)	2/28 (7.1%)	12/35(34.3%)	3/12 (25.0%)
No evidence of disease on MRI	17	3	1	1	10	2
Evidence of disease on MRI	9	3	2	1	2	1

In the adult cohort, disease origin was not significantly associated with recidivism (χ^2^ = 0.111; *p* = 0.946). Extension into mastoid antrum showed a trend toward higher recidivism, but this did not reach statistical significance (*p* = 0.076). Additionally, SIMD quintile was not associated with disease origin (χ^2^ = 6.994; *p* = 0.537) or with mastoid antrum extension (χ^2^ = 8.453; *p* = 0.390).

On univariate analysis, none of the examined variables (gender, smoking status and canal wall status [CWU vs. CWD], ossicular chain erosion, dehiscent facial nerve, socioeconomic status, origin of disease and extension into mastoid antrum) demonstrated a significant association with recidivism in the adult cohort. Age at operation, however, was inversely associated with recidivism (*p* = 0.004). When these variables were entered into the multivariate logistic regression model, age remained the only independent predictor of recidivism (*p* = 0.002). No other covariates reached statistical significance.

## Discussion

In our retrospective cohort of patients who underwent primary mastoidectomy for cholesteatoma between 2012 and 2020, we assessed residual/recurrence rates across SIMD quintiles. Our findings indicate that socioeconomic deprivation may be associated with an increased likelihood of residual or recurrent disease, with a more notable effect seen in the paediatric cohort. This suggests that deprivation may influence disease behaviour and postoperative outcomes differently in children compared with adults.

There is established evidence linking socioeconomic deprivation with a higher incidence of cholesteatoma.^4^ However, much less is known about whether deprivation also affects postoperative recurrence. This remains an important gap, given plausible pathways such as later presentation, greater initial disease burden, differences in access to follow-up care and higher rates of comorbidities in more deprived populations. Although socioeconomic deprivation has been associated with increased incidence of cholesteatoma, its relationship with disease severity at presentation was not observed in our cohort.^4^ Assessment of initial disease severity is further limited by the lack of routine clinical use of formal staging systems.^7^ Our study adds to this limited evidence base by focusing specifically on residual or recurrent disease and examining how deprivation may influence outcomes following primary mastoidectomy.

Based on our literature review, only a small number of studies have examined how socioeconomic or social factors relate to cholesteatoma outcomes. Aljehani *et al.* conducted a spot review of 190 patients and reported that recurrence was associated with factors such as lower educational level, hygiene-related practices, comorbidities and geographic region.^8^ Kennedy *et al*. reported higher rates of further surgery and residual disease among rural compared with urban patients in their single-surgeon adult cohort.^9^ Their analysis provides valuable early insight but does not directly address socioeconomic status, as rural–urban residence is not synonymous with deprivation. Although the authors considered insurance classification as a proxy for socioeconomic status, this is a limited surrogate and differs from direct measures of socioeconomic inequality. Patel *et al.* explored the prevalence of social determinants among adult cholesteatoma patients and found that stress and housing instability were common, but these factors were not associated with differences in presentation or outcomes.^10^ Their study relied on patient completed questionnaires, limiting generalisability.^10^

Our study contributes to this developing area by using the SIMD, a validated and widely used area-based measure of deprivation.^6^ Unlike single-factor proxies such as insurance status or individual questionnaire responses, SIMD provides a structured, composite assessment of deprivation across multiple domains, allowing more consistent comparison across populations. By examining recidivism through this lens, our work helps broaden the understanding of how wider social conditions may shape residual or recurrent disease.

The association between socioeconomic deprivation and recidivism was more evident in children, whereas adults showed weaker or non-significant trends. This may reflect differences in disease biology, as childhood cholesteatoma is typically more aggressive and more likely to recur.^1^ Children from deprived backgrounds may also face greater barriers to consistent follow-up, including transport difficulties, family pressure and lower caregiver health literacy. In adults, more stable engagement with long term care and differing disease characteristics may reduce the effect of deprivation on recurrence. The observed association between increasing age and lower recurrence risk may relate to shorter follow-up durations, competing comorbidities or less extensive disease when cholesteatoma first presents later in life.

In the paediatric cohort, recidivism rates were significantly higher in children from more deprived areas. Notably, SIMD quintile was not associated with extent of disease suggesting that socioeconomic deprivation influences recurrence through mechanisms other than initial disease severity. In adults, SIMD quintile was not associated with extent of disease and initial disease severity did not significantly predict residual or recurrent disease. These findings indicate that while disease extent strongly influences recidivism in children, other factors linked to deprivation may play a larger role, whereas in adults, this appears less dependent on both socioeconomic status and initial disease severity.

This study benefits from inclusion of all primary mastoidectomy cases for cholesteatoma within NHS Tayside over a 9-year period with rigorous case verification using operative documentation and the application of SIMD as a validated measure of area-based deprivation. These strengths support the reliability of our findings and provide a robust foundation for examining social influences on cholesteatoma outcomes.

Several limitations should be noted. This study includes all patients in the region who had primary mastoidectomy for cholesteatoma over nine years. However, the total number of patients, especially in the paediatric group, was small, which limited some analyses. In children, there were too few outcome events to perform multivariate analysis, so the link between socioeconomic deprivation and recurrence should be interpreted with caution. In adults, multivariate analysis was possible, but the lack of significant associations between most patient or surgical factors and recurrence may partly be due to limited sample size, which makes it harder to detect smaller effects across the SIMD quintiles. Larger multicentre studies would allow these relationships to be explored further. Additionally, as SIMD is an area-based measure of deprivation, it may not fully capture individual socioeconomic circumstances. The retrospective design limited application of standardised cholesteatoma classification (e.g., EAONO/JOS) and causal inference and unmeasured factors such as follow-up adherence and surgical variations may have influenced outcomes.^7^

Despite these limitations, the potential association between socioeconomic deprivation and recurrence, particularly among children, highlights important clinical implications. Strategies such as closer surveillance for high-risk groups, more frequent follow-up visits, enhanced family support, structured follow-up programmes and improved access to audiology and rehabilitation services may help reduce disparities. Future research should consider prospective multicentre designs incorporating individual-level socioeconomic data, detailed measures of disease severity and standardised follow-up protocols. Linkage with national datasets would facilitate larger cohorts and more robust modelling of predictors of residual or recurrent disease.

- Cholesteatoma is more prevalent and severe in socioeconomically deprived populations, but post-operative recurrence remains under-studied
- Socioeconomic deprivation, measured using the Scottish Index of Multiple Deprivation, is associated with increased residual or recurrent cholesteatoma in children
- Paediatric patients from more deprived backgrounds experience higher rates of recidivism following primary mastoidectomy
- This effect appears independent of initial disease characteristics, suggesting social factors may influence recurrence through mechanisms beyond disease severity
- No significant association between deprivation and residual/recurrence was observed in adults
- Consideration of socioeconomic factors may help inform post-operative surveillance strategies, particularly in children

## Conclusion

In summary, this study demonstrates that socioeconomic deprivation is associated with a higher likelihood of residual or recurrent cholesteatoma following primary mastoidectomy within NHS Tayside. This association appears more pronounced among paediatric patients. This effect appears independent of initial disease characteristics suggesting that social factors may influence recidivism through various other mechanisms beyond disease severity. These findings draw attention to an important but often under-recognised influence on postoperative outcomes and suggest that further work exploring the role of social determinants in cholesteatoma outcomes would be valuable.

## Figure 1 Long description

This bar chart shows residual or recurrent cholesteatoma rates in paediatric patients across SIMD quintiles. Recurrence is higher in more deprived groups and lowest in the least deprived quintile (SIMD 5), suggesting a socioeconomic gradient in paediatric outcomes.

Navigate back to Figure 1.

## Figure 2 Long description

This bar chart shows residual or recurrent cholesteatoma rates in adult patients across SIMD quintiles. Rates are relatively similar across all quintiles, with no clear association between socioeconomic status and recurrence.

Navigate back to Figure 2.

## Figure 3 Long description

This Kaplan–Meier curve shows time to recidivism in paediatric patients by SIMD quintile. There is a significant difference between groups (log-rank χ² = 13.05, p = 0.011), with more deprived quintiles demonstrating earlier and higher recurrence over time.

Navigate back to Figure 3.

## Figure 4 Long description

This Kaplan–Meier curve shows time to recidivism in adult patients by SIMD quintile. There is no significant difference between groups (log-rank χ² = 1.86, p = 0.76), indicating similar outcomes across socioeconomic groups.

Navigate back to Figure 4.

## Table 1 Long description

The table summarizes baseline characteristics, surgical factors, and post-operative follow-up for 59 children overall and by SIMD quintile (SIMD 1 to SIMD 5). Overall, 59.3% were male and 40.7% female, with the highest male proportion in SIMD 3 (85.7%) and SIMD 4 (80.0%). Canal wall-up technique was used in 81.4% overall (ranging from 73.3% in SIMD 4 to 92.3% in SIMD 5), while canal wall-down was 18.6%. Primary disease location was most often the attic (66.1% overall), with pars tensa at 23.7% and both sites at 10.2%. Extension into the mastoid antrum was common (79.7% overall), reaching 100.0% in SIMD 2, and ossicular chain erosion was also frequent (83.1% overall), including 100.0% in SIMD 1 and SIMD 3. Dehiscent facial nerve was rare overall (3.4%) and occurred only in SIMD 1 (20.0% within that group). Recurrence after primary surgery occurred in 39.0% overall, varying widely by SIMD group from 0% in SIMD 3 to 66.7% in SIMD 4. Median follow-up was 74.0 months overall (interquartile range 45.5 to 103.0), longest in SIMD 4 at 101.0 months and shortest in SIMD 5 at 48.0 months. Planned second-look surgery was performed in 40.7% overall, and among those second looks the findings were 10 with no disease, 5 with recurrence, and 9 with residual disease; post-operative MRI was done in 64.4% overall, with 24 showing no evidence of disease and 14 showing evidence of disease.

Navigate back to Table 1.

## Table 2 Long description

The table summarizes adult patient demographics, disease and surgical features, and post-operative follow-up for 125 patients overall and by SIMD quintile (1 to 5). Overall, 51.2 percent were male and 48.8 percent female; 30.4 percent smoked at surgery, 64.8 percent were lifelong non-smokers, and 4.8 percent were ex-smokers. Canal wall-down surgery was more common than canal wall-up (69.6 percent vs 30.4 percent), with the highest canal wall-down proportion in SIMD 2 (77.3 percent). Primary disease was usually in the attic (84.8 percent overall), while pars tensa involvement was less common (11.2 percent) and both sites were rare (4.0 percent, occurring only in SIMD 3 and 4). Extension into the mastoid antrum occurred in 66.4 percent overall and was highest in SIMD 4 (77.1 percent). Ossicular chain erosion was frequent (72.8 percent overall) and dehiscent facial nerve was reported in 19.2 percent overall, varying from 10.7 percent in SIMD 1 to 25.0 percent in SIMD 3. Recurrence after primary surgery occurred in 24.0 percent overall, ranging from 8.3 percent in SIMD 5 to 31.8 percent in SIMD 2. Median follow-up was 60.0 months overall (interquartile range 47.0 to 87.0), longest in SIMD 1 (74.5 months) and shortest in SIMD 5 (44.5 months). Planned second-look surgery was done in 7.2 percent overall (none in SIMD 2), and among those second-looks most findings were residual disease rather than recurrence. Post-operative MRI was performed in 20.8 percent overall, with no evidence of disease in 17 of 26 scans and evidence of disease in 9 of 26; MRI use was highest in SIMD 4 (34.3 percent).

Navigate back to Table 2.
